# Static and dynamic resting-state brain activity patterns of table tennis players in 7-Tesla MRI

**DOI:** 10.3389/fnins.2023.1202932

**Published:** 2023-07-14

**Authors:** Yuyang Li, Mengqi Zhao, Yuting Cao, Yanyan Gao, Yadan Wang, Bing Yun, Le Luo, Wenming Liu, Chanying Zheng

**Affiliations:** ^1^Key Laboratory of Medical Neurobiology of Zhejiang Province, Interdisciplinary Institute of Neuroscience and Technology, School of Medicine, Zhejiang University, Hangzhou, China; ^2^School of Psychology, Zhejiang Normal University, Jinhua, China; ^3^Key Laboratory of Intelligent Education Technology and Application of Zhejiang Province, Zhejiang Normal University, Jinhua, China; ^4^Key Laboratory for Biomedical Engineering of Ministry of Education, College of Biomedical Engineering and Instrument Science, Zhejiang University, Hangzhou, China; ^5^College of Information and Electronic Technology, Jiamusi University, Jiamusi, China; ^6^Department of Public Physical and Art Education, Zhejiang University, Hangzhou, China; ^7^Hangzhou Wuyunshan Hospital, Hangzhou, China; ^8^Department of Sport Science, College of Education, Zhejiang University, Hangzhou, China

**Keywords:** table tennis, motor training, resting-state fMRI, intrinsic brain activity, visuomotor responses, percentage amplitude fluctuation, dynamic amplitude of low frequency fluctuation, dynamic functional connectivity

## Abstract

Table tennis involves quick and accurate motor responses during training and competition. Multiple studies have reported considerably faster visuomotor responses and expertise-related intrinsic brain activity changes among table tennis players compared with matched controls. However, the underlying neural mechanisms remain unclear. Herein, we performed static and dynamic resting-state functional magnetic resonance imaging (rs-fMRI) analyses of 20 table tennis players and 21 control subjects using 7T ultra-high field imaging. We calculated the static and dynamic amplitude of low-frequency fluctuations (ALFF) of the two groups. The results revealed that table tennis players exhibited decreased static ALFF in the left inferior temporal gyrus (lITG) compared with the control group. Voxel-wised static functional connectivity (sFC) and dynamic functional connectivity (dFC) analyses using lITG as the seed region afforded complementary and overlapping results. The table tennis players exhibited decreased sFC in the right middle temporal gyrus and left inferior parietal gyrus. Conversely, they displayed increased dFC from the lITG to prefrontal cortex, particularly the left middle frontal gyrus, left superior frontal gyrus-medial, and left superior frontal gyrus-dorsolateral. These findings suggest that table tennis players demonstrate altered visuomotor transformation and executive function pathways. Both pathways involve the lITG, which is a vital node in the ventral visual stream. These static and dynamic analyses provide complementary and overlapping results, which may help us better understand the neural mechanisms underlying the changes in intrinsic brain activity and network organization induced by long-term table tennis skill training.

## 1. Introduction

Table tennis is a fast-paced, open skill sport invented in the late 19th century (Chen et al., 2019). The lightweight racket and hollow ball can easily be handled by most people, including older adults and young kids (Faber et al., 2021; Ke et al., 2021). Table tennis is characterized by high speed, multiple changes, and strong confrontation. Table tennis can be reportedly used as an exercise intervention to improve cognition in older adults and alleviate depression (Jeoung, 2014; Buzzelli and Draper, 2020; Ke et al., 2021). Athletes in different domains have different intrinsic brain activities depending on sports characteristics (Raichlen et al., 2016; Tan et al., 2016; Yang et al., 2020). For example, soccer players have stronger amplitude of low-frequency fluctuations (ALFF) in the right inferior frontal gyrus, which plays a crucial role in inhibition, compared to competitive aerobics athletes (Shi et al., 2020). Since table tennis requires fast responses and visual motor coordination, after years of deliberate practice, table tennis players can quickly and accurately initiate a targeted motor response without extensive cognitive processing, thus having a shorter visual motor response time than non-athletes (Hülsdünker et al., 2018, 2019). Therefore, visuomotor reaction time can be used to reflect the cognition and skill performance of players (Patel et al., 2014; Verschueren et al., 2020; Roberts et al., 2022). This superior behavior of table tennis players may be related to deliberate training, which results in unique intrinsic brain activity patterns (Cheron et al., 2016; Perrey and Besson, 2018; Moore et al., 2022).

Athletes are good human models for studying neurological adaptations owing to long time (years) of deliberate practice and continuous learning in a specific domain (Ericsson and Lehmann, 1996; Nakata et al., 2010; Yin et al., 2021). Both processes alter brain functional activity, which is associated with optimal athletic behavioral performance (Ericsson, 2013). Ericsson believes that deliberate practice allows athletes to rapidly encode and retrieve information from long-term working memory for better behavior control and performance (Ericsson and Kintsch, 1995). Further, three neurobiological hypotheses aim to explain the underlying mechanism. The neural efficiency hypothesis suggests that athletes tend to display more automatic and spatially localized brain activity, which is less intense than that of non-athletes (Li and Smith, 2021). This is achieved by selectively engaging only the neural networks necessary to perform a specific task in an optimal manner (Ericsson, 2007). According to the second, transient hypofrontality hypothesis, the superior performance of athletes can be attributed to decreased brain activity in the frontal lobe (Dietrich, 2003; Dietrich, 2006; Filho et al., 2021). Finally, the neural proficiency hypothesis posits that skilled performance requires not only the downregulation of task-irrelevant neural networks but also the upregulation (i.e., neutral recruitment) of task-relevant brain networks (Bertollo et al., 2016; Filho et al., 2022; Gao et al., 2023). However, the neuronal activity alterations associated with athletic training are diverse and cannot fully be explained by any single hypothesis.

Resting-state functional magnetic resonance imaging (rs-fMRI) is widely used to study intrinsic brain activity (Hu et al., 2022), as it reflects spontaneous brain activity by blood oxygen level-dependent (BOLD) signals in the unstimulated state (Biswal et al., 1995; Chen et al., 2023). ALFF and functional connectivity (FC) are two indicators commonly used in rs-fMRI (Di et al., 2012; Chang et al., 2018; Bertollo et al., 2020; Cao et al., 2021; Zhang et al., 2021, 2022). ALFF is a stable biomarker that reflects local spontaneous activity and measures the crude signal intensity of local brain areas in low-frequency (0.01–0.08 Hz; Yang et al., 2007; Zang et al., 2007). Current studies regarding spontaneous neural activity in the brain of table tennis players focus on the conventional frequency band, i.e., 0.01–0.08 Hz (Shi et al., 2020; Roberts et al., 2022). However, single-band results lack frequency specificity (Buzsáki and Draguhn, 2004; Gao et al., 2015). Therefore, we calculated ALFF in the regular frequency band (0.01–0.08 Hz) and in the slow-4 and slow-5 bands (0.027–0.073 and 0.01–0.027 Hz, respectively; Zuo et al., 2010). Various brain regions do not work in isolation but cooperate with each other. FC can respond to functional integration between anatomically separate brain regions by calculating the temporal correlation of the fMRI time series between different regions (van den Heuvel and Hulshoff Pol, 2010; Mohanty et al., 2020). Therefore, we consequently also used FC analysis in this study.

Furthermore, existing rs-fMRI analysis methods mostly consider static brain activity during the scan in athlete-related studies. However, the brain is a highly dynamic system with erratic neural activities and rapidly changing neural interactions, indicating that brain activities alter over time (Han et al., 2011). Consequently, information may be lost if only the average state of brain activity in a time series is used to represent the intensity of neuron activity. Therefore, combined with static rs-fMRI calculations and a sliding window, dynamic fMRI has been proposed to describe the temporal variability of brain activities (Sakoğlu et al., 2010; Liang et al., 2021). Dynamic changes in resting-state brain activity are associated with cognition and behavioral traits, and dynamic rs-fMRI is a more sensitive biomarker in some cases than static rs-fMRI. For example, some studies have reported that dynamic functional connectivity (dFC) can more sensitively capture task-based phenotypes (e.g., processing speed or fluid intelligence scores) than static FC, whereas several others have reported that dynamic brain connectivity is a better predictor of PTSD than static connectivity (Jin et al., 2017; Liégeois et al., 2019; Liang et al., 2021). Therefore, herein, we performed static and dynamic joint analysis of rs-fMRI. In addition, we used the percentage amplitude of fluctuation (PerAF) to measure dynamic rs-fMRI indicators instead of the commonly used coefficient of variation (CV; Tijhuis et al., 2021). PerAF is an index of percentage signal change that is used to measure BOLD signal fluctuations during rs-fMRI in a single-voxel level and demonstrates good test–retest reliability according to previous studies (Jia et al., 2020; Gao et al., 2022). Therefore, we used PerAF to calculate the signal collected by each window to describe the percentage fluctuations of dynamic rs-fMRI indicators.

Thus, we performed static and dynamic joint analysis of rs-fMRI and calculated the static ALFF (sALFF), dynamic ALFF (dALFF), static functional connectivity (sFC), and dFC to comprehensively study the changes in intrinsic brain activity related to visuomotor responses in table tennis players. Notably, we used a 7T ultra-high field magnetic resonance system (Siemens Healthcare, Erlangen, Germany) to obtain rs-fMRI data with high spatial and temporal signal-to-noise ratio (Triantafyllou et al., 2005).

We hypothesized that (i) combined static and dynamic rs-fMRI analyses could yield complementary or overlapping results about the changes in intrinsic brain activity among table tennis players; (ii) these changes would occur in visual, sensorimotor and executive function areas; and (iii) these changes would be associated with superior visuomotor responses of table tennis players than controls.

## 2. Materials and methods

### 2.1. Participants

For 7T MRI scanning, we recruited 20 student table tennis players (aged 22.2 ± 2.75 years, 12 men, and 15.6 ± 2.11 years of education) and 21 healthy control participants who were matched in gender, age, and educational level (aged 22.62 ± 2.41 years, 12 men, and 16.62 ± 2.15 years of education; Table 1). All student athletes were Chinese national first-class table tennis players (“Athlete Technical Grade Standard” issued by the General Administration of Sports of China was considered the evaluation principle) with >6 years of training experience, whereas none of the healthy control participants had any sports specialties or hobbies. All the participants exhibited normal or corrected to normal vision and were right-handed with no history of neurological and psychiatric diseases. All experimental procedures were approved by the research ethics review committee of Wuyunshan Hospital ([2021] Clinical Research Medical Ethics Review No. 004) and conducted in accordance with the Declaration of Helsinki. All the participants provided informed written consent before the experiment and no drugs were administered during the experiment that could alter mental status, cognition, or perception.

**Table 1:** Demographic characteristics of the athletes and controls.

	Athletes (*n* = 20)	Controls (*n* = 21)	$\chi^{2}$ or *t*	*p* value
Age (years)	22.2 ± 2.75	22.62 ± 2.41	0.6794	0.5009^a^
Sex (female/male)	8/12	9/12	0	>0.9999^b^
BMI	22.23 ± 4.93	21.85 ± 2.99	0.3060	0.7612^a^
Education (years)	15.6 ± 2.11	16.62 ± 2.15	1.494	0.1431^a^
Training time (h/week)	7.13 ± 3.69	NA	NA	NA
Duration (years)	12.15 ± 2.83	NA	NA	NA
Mean FD (mm)	0.06 ± 0.02	0.06 ± 0.02	0.3933	0.6963^a^

Data are expressed as mean values ± SD; BMI, body mass index; SD, standard deviation; FD, frame displacement.

a *p* value obtained by two-sample *t*-test.

b *p* values obtained by Chi-squared test.

### 2.2. MRI data acquisition

MRI scanning experiments were performed using Nova Medical 32-channel array head coils in a 7T whole-body magnetic resonance system (Siemens Healthcare, Erlangen, Germany) and included rs-fMRI and structural image scanning. rs-fMRI data were acquired using an echo-planar imaging sequence with 1.5-mm isotropic resolution with the following parameters: repetition time (TR) = 2,000 ms, echo time (TE) = 20.6 ms, flip angle = 70°, field of view (FOV) = 228 mm × 228 mm, number of slices = 90, and total volume = 160. Structural images were acquired using the MP2RAGE sequence with 0.7- mm isotropic resolution with the following parameters: TR = 2,300 ms, TE = 3.09 ms, flip angle = 7°, and FOV = 225 mm × 225 mm. During the scan, the participants were asked to close their eyes, remain awake, and stay as still as possible.

### 2.3. MRI data preprocessing

Resting-state functional images were preprocessed using RESTplus V1.24^1^ (Jia et al., 2019) based on MATLAB 2017a (Mathworks, Natick, MA, United States).

The first five time points of resting-state fMRI data were discarded owing to the brief adaptation period required when a participant first enters the scanner and the instability of the initial MRI signal, leaving 155 time points per participant for further analysis (Liu et al., 2022). Head motion correction was performed, and the participant was excluded if their head movement exceeded 2.5 mm in any direction or 2.5° in any angle (Yan et al., 2013). Individual structural images were coregistered with the mean functional images and segmented into gray matter, white matter, cerebrospinal fluid, skull, extracerebral, and soft tissue using NewSegment tool in RESTplus V1.24. Coregistered structural images were normalized to the standard Montreal Neurological Institute space. For subsequent statistical analysis, a Gaussian kernel with 6 mm full width at half height (FWHM) was used for spatial smoothing; the linear trend of the time series was subsequently eliminated. To reduce the effect of physiological noise, the following interfering covariates were regressed: 24 head motion parameters, white matter signal, and cerebrospinal fluid signal (Friston et al., 1996). Finally, the FC was calculated after temporal band-pass filtering (0.01 to 0.08 Hz). Note that ALFF was calculated without filtering during preprocessing. No participants were excluded from the final dataset for analysis.

### 2.4. sALFF in different frequency bands and dALFF computation

**Figure 1:**
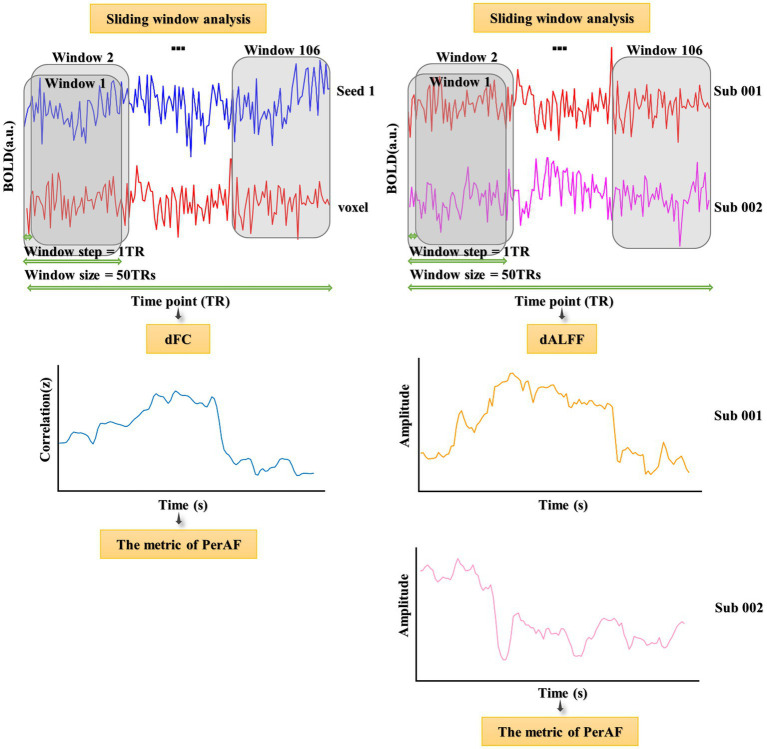
Flowchart of dALFF and dFC analyses and illustration of their temporal variability.

Three frequency bands (conventional frequency, slow-4, and slow-5 bands at 0.01–0.08, 0.027–0.073, and 0.01–0.027 Hz, respectively) of sALFF values were calculated using RESTplus V1.24. Specifically, the preprocessed time series was converted to a frequency domain using a fast Fourier transform, and the power spectrum was acquired. Average values were then calculated (average value of the square root of the power) at different amplitudes to obtain the ALFF values. Finally, the ALFF of each voxel was divided by the global average ALFF value to achieve standardization (Zang et al., 2007). The sliding window method was used to evaluate the dALFF using the temporal dynamic analysis module of RESTplus V1.24 (Jia et al., 2019; Gao et al., 2021; Figure 1). According to recommendations, the minimum window length should be >1/fmin, where fmin indicates the minimum frequency of time courses (Leonardi and Van De Ville, 2015). Based on previous studies, we chose a rectangular window with a length of 50 TRs and a moving step of 1 TR, producing 106 segmented windows (He et al., 2019; Fateh et al., 2020). The ALFF map was then computed for each window. Finally, based on a series of ALFF maps in all the windows, the PerAF corresponding to each voxel was computed, producing a dALFF map (Li et al., 2019; Gao et al., 2022). To verify the reliability of the metric of PerAF, the CV of the dALFF plot across windows was also calculated (Gao et al., 2022; Supplementary Figure S1).

### 2.5. Voxel-wised sFC and dFC computation

After calculating sALFF and dALFF, the groups were compared to identify several significantly differing brain regions. These were selected as seeds to perform voxel-wised sFC and dFC calculations. sFC and dFC values were calculated at the voxel level using RESTplus V1.24. When calculating sFC, the Person correlation coefficient (*r* value) was calculated between the average time series of the seed region and every voxel of the whole brain (Bandettini et al., 1993; Gao et al., 2019; Mitra et al., 2023; Shahsavarani et al., 2023; Thippabhotla et al., 2023). Next, a Fisher’s *r* to *z* translation was applied to convert the *r* value to a normalized *z* value. The whole-brain FC map of each seed region could then be obtained. To calculate dFC, the same sliding window parameters were used as for dALFF (Figure 1). For every sliding window, a whole-brain *z* values FC map could be obtained between the mean time series of the seed region and every voxel of the whole brain (Gao et al., 2022). Notably, the number of FC probability maps was equal to the number of sliding windows. Finally, based on a range of *z* values FC maps in all windows, the PerAF of each voxel was calculated to obtain a dFC map. To verify the reliability of the metric of PerAF, the CV of the dFC map across windows was also calculated (Gao et al., 2022; Supplementary Figure S2).

### 2.6. PerAF calculation

The PerAF is one index of percentage signal change that is used to measure the BOLD signal fluctuations during rs-fMRI (Jia et al., 2020). Herein, we used PerAF to measure the percentage of dALFF and dFC change by converging the dALFF and dFC values of all the windows. The PerAF of each voxel is calculated as:


(1) $$PerAF=\frac{1}{n}\overset{n}{\underset{i=1}{\sum }}\left|\frac{X_{i}-\mu }{\mu }\right|\times 100\%$$

(2) $$\mu =\frac{1}{n}\overset{n}{\underset{i=1}{\sum }}X_{i}$$

During the calculation of dALFF, *X_i_* refers to the ALFF value of the *i_th_* window. During the calculation of dFC, *X_i_* is the *z* value of the *i_th_* window, and the *z* value is converted from the *r* value of the mean FC in *i_th_* windows using Fisher’s *r* to *z* translation. *n* is the number of sliding windows, and *μ* is the average of ALFF or *z* values of the *n* sliding windows.

### 2.7. Visual movement experimental procedure

**Figure 2:**
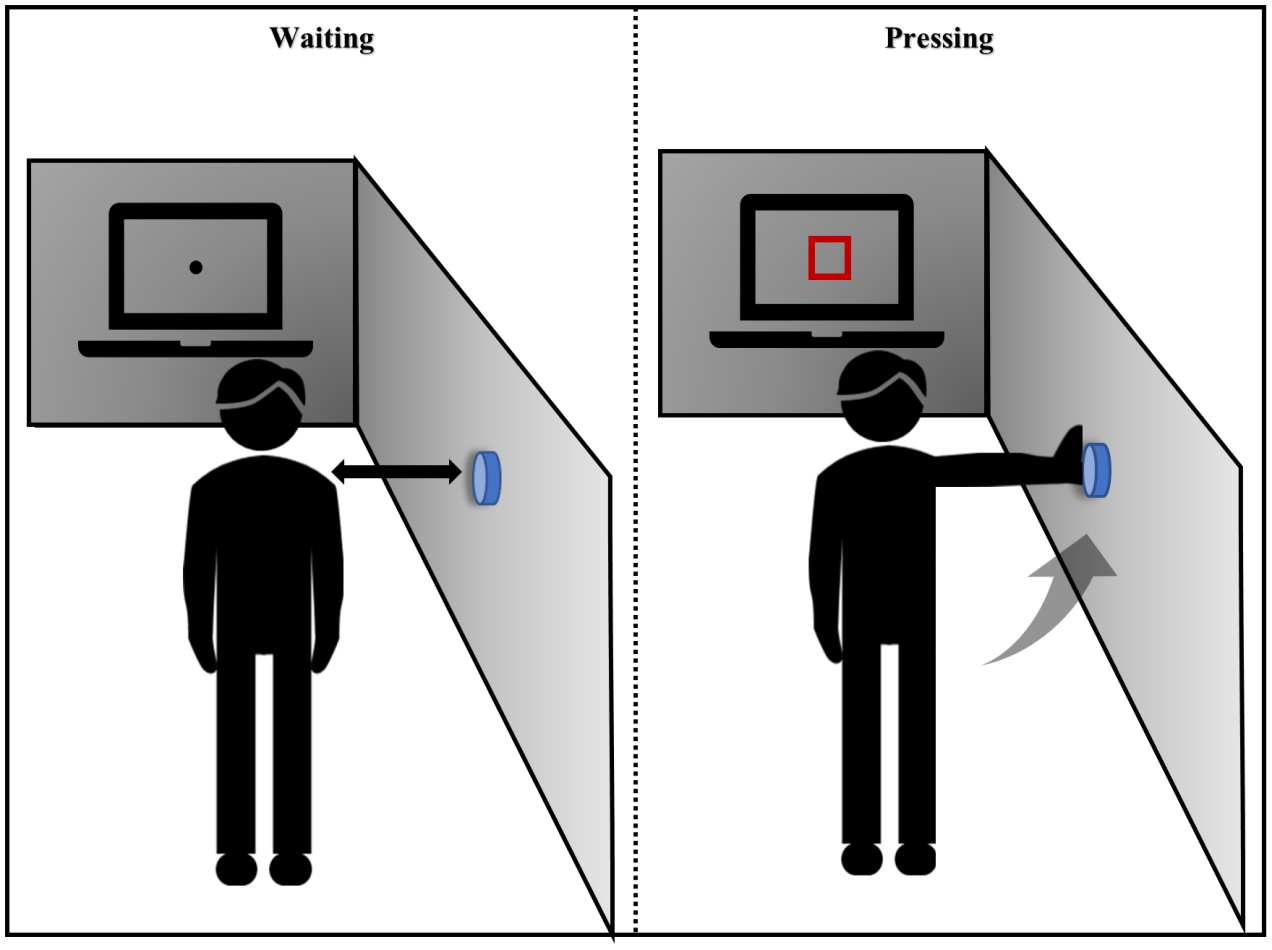
Instructions for the visual movement experiment.

The experimental procedure was adapted from the Eye-Hand coordination paradigm of the Nike Sensory Station assessments and was written using Psychophysics Toolbox based on MATLAB (MathWorks, Natick, MA, United States; Erickson et al., 2011; Farshchian et al., 2018; Figure 2). Stimuli were shown on a linearized monitor (1920 × 1,080 resolution, 100-Hz refresh rate) and drawn against a gray (56 cd/m^2^) background (Liu et al., 2022). The participants stood with their feet shoulder-width apart at an arm’s length from the wall and 1 m from the horizontal screen. The researcher adjusted the height of the buttons on the wall to the level of the participant’s shoulders. The visuomotor stimulus comprised 10 trials; each trial included a waiting and pressing state, and the participants were asked to focus on the screen the entire time. In the waiting state, a black fixation point appeared at the center of the screen. At this time, the participants kept their arms hanging down naturally and close to their thighs. After 5 s, the black spots disappeared and a red box immediately appeared in the middle of the screen. The participants were asked to raise their hands and press the button on the wall when they saw the red box, and then immediately returned to the waiting state. This cycle was repeated 10 times. The participants could familiarize themselves with the experimental operation before the formal experiment started. The reaction time elapsed from the appearance of the red box until the participant pressed the button was recorded.

### 2.8. Statistical analysis

The demographic information of all the participants was statistically analyzed using GraphPad prism 8. Chi-square test was performed to compare sex, and two-sample *t*-test was used for to compare age, years of education, and mean frame-wise displacement (FD) parameters. The threshold of statistical significance was established at a level of *p* < 0.05.

Statistical analysis of the four kinds of rs-fMRI for the athlete and control groups was performed using RESTplus V1.24, and two-sample *t*-test was performed for the analysis. To avoid the effect of covariates, regression analysis was performed for age, gender, education, and body mass index. For the results of two-sample *t*-test using Gaussian random field (GRF) theory for multiple comparisons correction, the voxel level threshold was *p* < 0.01 and the cluster level threshold *was p* < 0.05. The magnitude d effect size and confidence intervals were calculated.

For behavioral data, we used mean ± 2SD to exclude outliers and then calculated the average individual reaction time as behavioral indicators. SPSS 20 (IBM, United States) was used to evaluate the correlation between the mean reaction time and MR variables (sALFF, sFC, dALFF, and dFC) *via* Pearson’s correlation analysis. The mean reaction time differences between the groups were calculated using two-sample *t*-test. Differences or correlations were considered statistically significant if *p* was <0.05. The magnitude d effect size and confidence intervals were calculated.

## 3. Results

### 3.1. Demographic characteristics

The demographic characteristics of the athlete and control groups are shown in Table 1. No significant differences in age (*t* = 0.6794, *p* = 0.5009), sex ( $\chi^{2}$ = 0, *p* > 0.9999), BMI (*t* = 0.3060, *p* = 0.7612), education (*t* = 1.494, *p* = 0.1431), and mean FD (*t* = 0.3933, *p* = 0.6963) were observed between the athletes and controls.

### 3.2. Differences in sALFF and dALFF between the athlete and control groups

**Figure 3:**
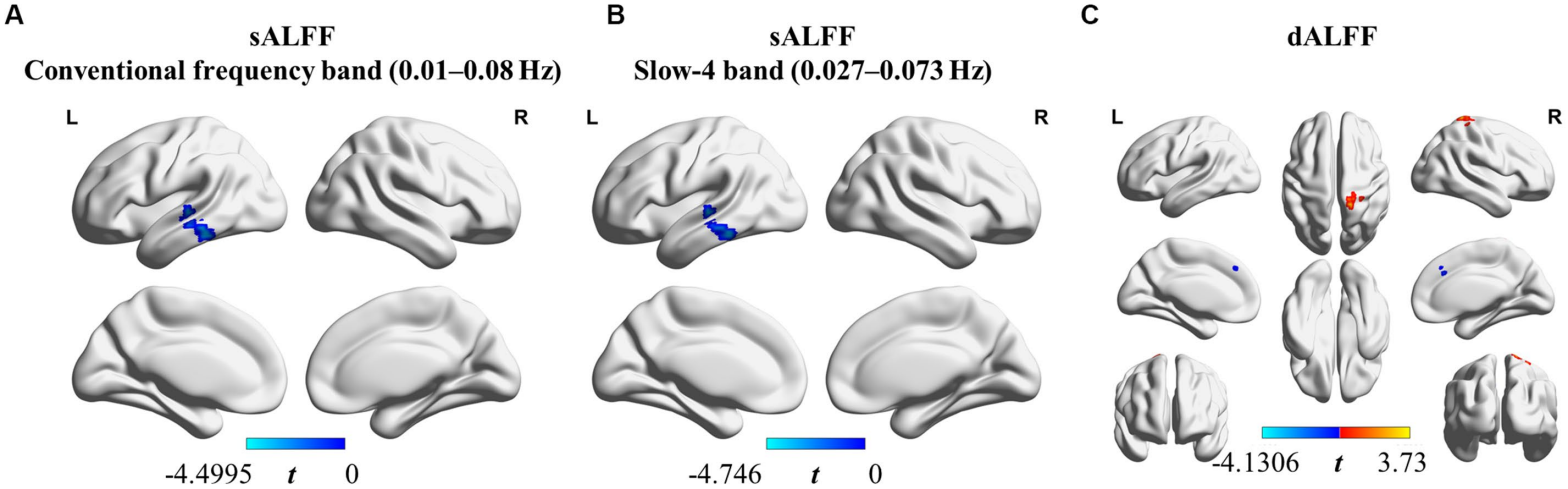
sALFF (in two frequency bands) and dALFF differences between athletes and controls. **(A)** sALFF differences in the conventional frequency band (0.01–0.08 Hz) between athletes and controls; **(B)** sALFF differences in the slow-4 band (0.027–0.073 Hz) between athletes and controls; **(C)** dALFF differences between athletes and controls. Two-sample *t*-test, voxel-level *p* < 0.01, cluster-level *p* < 0.05, GRF correction; Blue area: Athletes < Controls. Red area: Athletes > Controls.

We found that sALFF and dALFF provided complementary information when studying differences in brain activity between athletes and controls. Three frequency bands (conventional frequency, slow-4, and slow-5 bands at 0.01–0.08, 0.027–0.073, and 0.01–0.027 Hz, respectively) were selected to calculate sALFF. Two-sample *t*-test results revealed that athletes exhibited smaller sALFF values in the left inferior temporal gyrus (lITG) when compared with those in controls in the traditional and slow-4 frequency bands; the *t*-values of the two frequency bands were different (Table 2; Figures 3A,B; Supplementary Figure S3, GRF correction, voxel *p* < 0.01 cluster *p* < 0.05). However, significant differences were not present between the groups in the slow-5 frequency band.

**Table 2:** sALFF differences in two frequency bands and dALFF differences between athletes and controls.

Brain region	Cluster size (in voxels)	Coordinate (x, y, z)			Peak *t* value	Effect size	Confidence intervals (95%)
**sALFF Conventional frequency band (0.01–0.08 Hz)**							
Left inferior temporal gyrus	83	−66	−39	−21	−4.4995	−1.4058	[0.7118, 2.0854]
**sALFF Slow-4 band (0.027–0.073 Hz)**							
Left inferior temporal gyrus	74	−66	−39	−21	−4.746	−1.4828	[0.7805, 2.1701]
**dALFF**							
Right middle cingulate and paracingulate gyri	18	3	27	36	−4.1306	−1.2906	[0.6079, 1.9593]
Right postcentral gyrus	26	27	−39	72	3.73	1.1654	[0.4948, 1.8235]

sALFF, static amplitude of low-frequency fluctuation; dALFF, dynamic amplitude of low-frequency fluctuation; *t* < 0 means lower sALFF or dALFF value of athletes than controls, *t* > 0 means higher sALFF or dALFF value of athletes than controls. Two-sample *t*-test, voxel-level *p* < 0.01, cluster-level *p* < 0.05, GRF correction.

In addition, we used PerAF to measure the dALFF to represent the BOLD signal fluctuations. Athletes had markedly higher dALFF values in the right postcentral gyrus (rPoCG) than those in the controls, whereas dALFF values in the right middle & paracingulate gyri (rMCC) were reduced in athletes (Table 2; Figure 3C; GRF correction, voxel *p* < 0.01 cluster *p* < 0.05). All effect size statistics are expressed as Cohen’s d, which were > 0.8, indicating that our results are stable and reliable (Seymour et al., 2016).

### 3.3. Differences in static and dynamic voxel-wised FC between athlete and control groups

**Figure 4:**
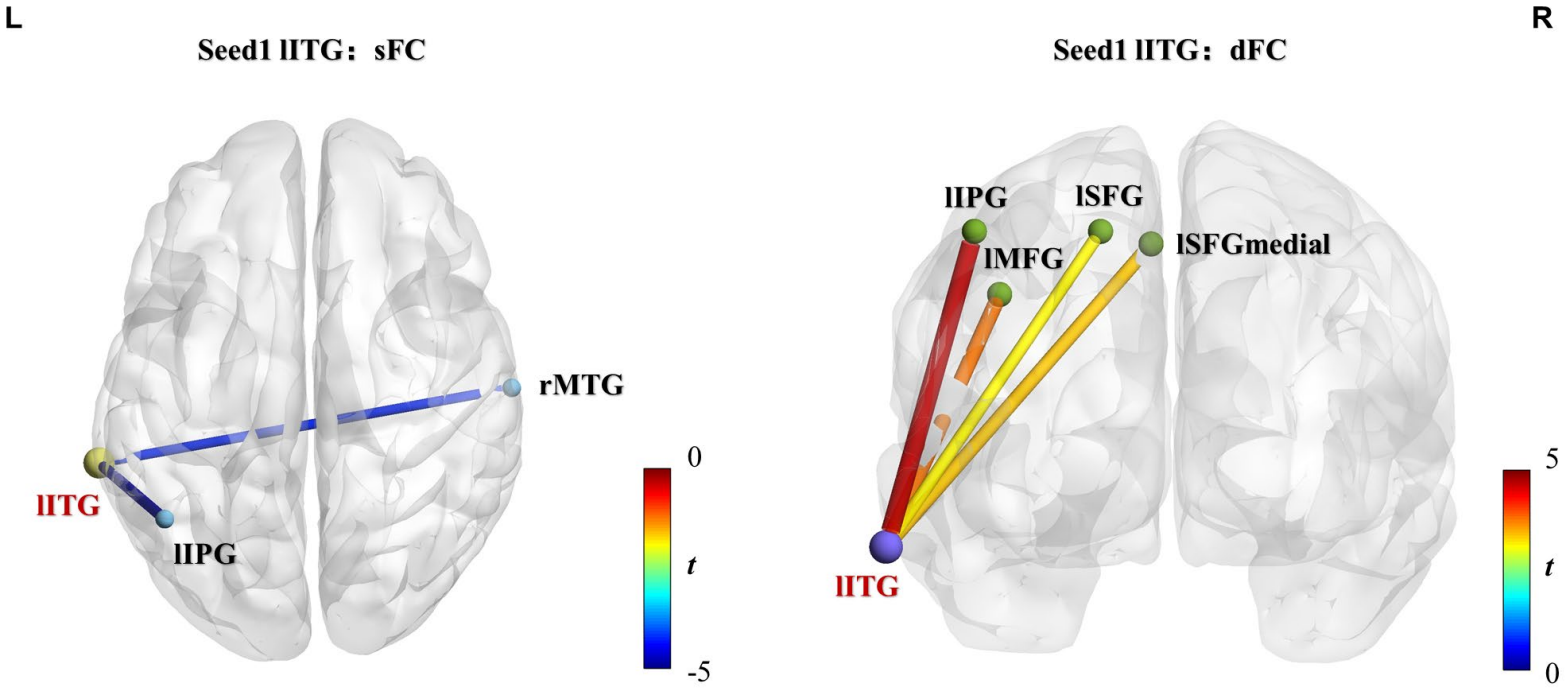
lITG seed-based static and dynamic FC differences between athletes and controls. Significantly increased sFC or dFC in the athlete group is shown in warm colors, whereas significantly decreased sFC or dFC is indicated in cold colors. The line colors represent t-values. Two-sample *t*-test, voxel-level *p* < 0.01, cluster-level *p* < 0.05, GRF correction. sFC, static functional connectivity; dFC, dynamic functional connectivity; lITG, left inferior temporal gyrus; lIPG, left inferior parietal gyrus; rMTG, right middle temporal gyrus; lMFG, left middle frontal gyrus; lSFGmedial, left superior frontal gyrus-medial; lSFG, left superior frontal gyrus-dorsolateral.

Three brain regions, lITG, rMCC, and rPoCG, were selected as seeds, which were used to perform whole-brain voxel-wised sFC and dFC calculations. The brain regions showing significantly different static and dynamic functional connections between the two groups are shown in Figure 4 or Supplementary Figure S4 and the results are summarized in Table 3 (GRF correction, voxel *p* < 0.01, cluster *p* < 0.05).

**Table 3:** Static and dynamic voxel-wised FC (using the left inferior temporal gyrus, right middle cingulate and paracingulate gyri, and right postcentral gyrus as seed regions) differences between athletes and healthy controls.

Seed	Brain region	Cluster size (in voxels)	Coordinate (x, y, z)			Peak *t* value	Effect size	Confidence intervals (95%)
Left inferior temporal gyrus	sFC							
	Right middle temporal gyrus	95	66	−15	−12	−4.2959	−1.3422	[0.6544, 2.0158]
	Left inferior parietal gyrus	160	−45	−57	48	−4.763	−1.4882	[0.7855, 2.1759]
	dFC							
	Left middle frontal gyrus	13	−39	24	39	3.792	1.1848	[0.5121, 1.8444]
	Left superior frontal gyrus-medial	7	−3	45	51	3.4448	1.0763	[0.4132, 1.7273]
	Left superior frontal gyrus-dorsolateral	6	−15	24	54	3.1947	0.9982	[0.3415, 1.6434]
	Left inferior parietal gyrus	25	−45	−54	54	4.5277	1.4146	[0.7198, 2.0951]
Right middle cingulate and paracingulate gyri	–	–	–			–		
Right postcentral gyrus	–	–	–			–		

dFC, dynamic functional connectivity; sFC, static functional connectivity; *t* < 0 means lower FC value of athletes than of controls; *t* > 0 means higher FC value of athletes than of controls. Two-sample *t*-test, voxel-level *p* < 0.01, cluster-level *p* < 0.05, GRF correction.

Group differences of sFC and dFC were only present when the lITG was used as a seed, whereas the rMCC and rPoCG seed regions did not yield significant results in sFC and dFC analysis. When the lITG was selected as the seed for sFC analysis, the athlete group exhibited a significantly decreased sFC in the right middle temporal gyrus (rMTG) and left inferior parietal gyrus (lIPG). From the perspective of dFC, athletes exhibited increased dFC from the lITG to the left middle frontal gyrus (lMFG), left superior frontal gyrus-medial (lSFGmedial), left superior frontal gyrus-dorsolateral (lSFG), and lIPG compared with that in controls. These results were corrected using GRF correction (voxel *p* < 0.01 and cluster *p* < 0.05). The effect sizes are expressed using Cohen’s *d* values, which were > 0.8, thus indicating that our results are stable and reliable.

### 3.4. Correlation analysis results of visuomotor reaction time and resting state functional indices

**Figure 5:**
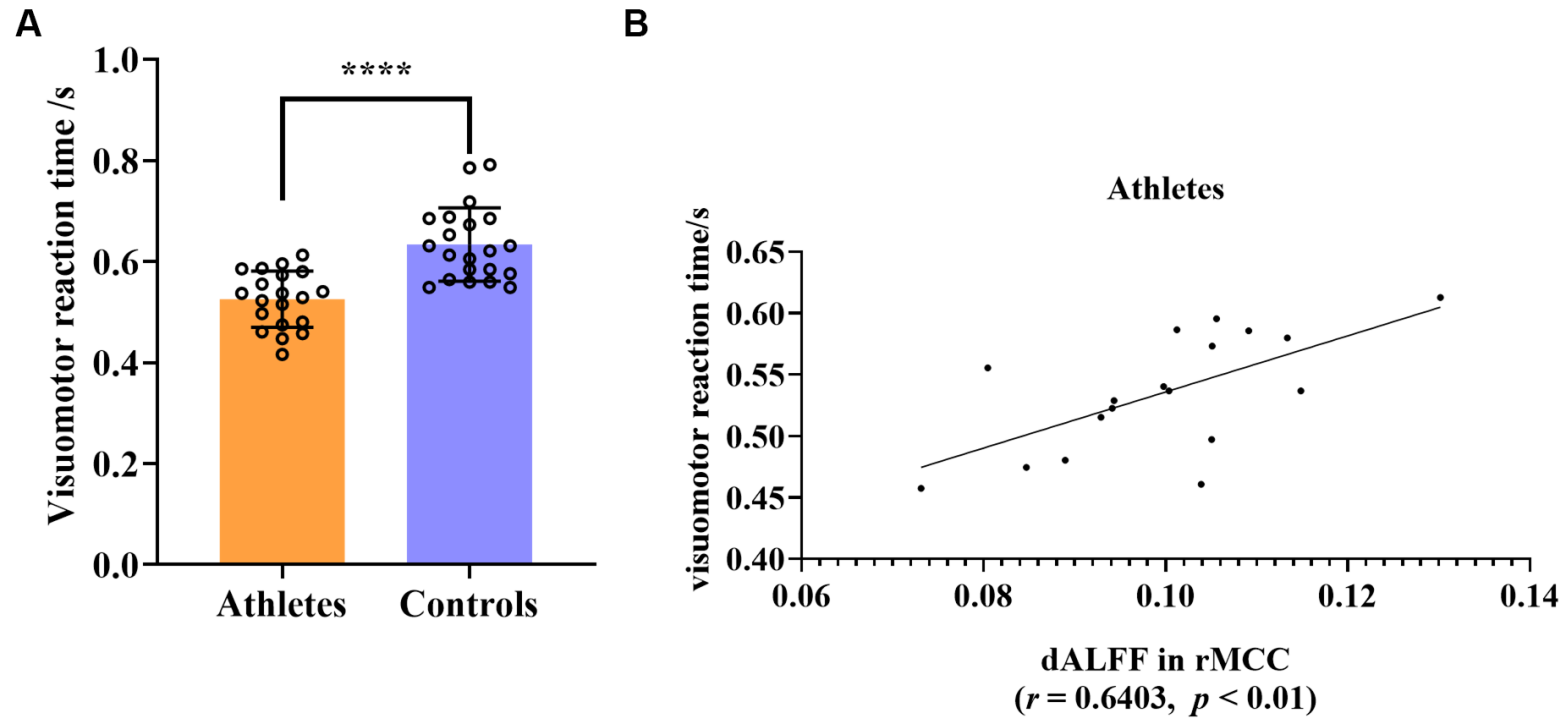
**(A)** Group differences in visuomotor reaction time between athletes and controls. **(B)** Significant correlation between visuomotor reaction time and dALFF value of rMCC in athletes. ****p < 0.0001; rMCC, right middle cingulate and paracingulate gyri.

The visuomotor reaction time of the athlete group was significantly shorter than that of the control group (mean ± 2SD, athletes: 0.5252 ± 0.1085, controls: 0.6336 ± 0.1414, *t* = 5.351, *p* < 0.0001, *d* effect size = 1.6719, confidence intervals = [0.9486, 2.3793], *df* = 39, Figure 5A). The signal of each participant of the rs-fMRI index was extracted separately, and the correlation with the individual average visuomotor reaction time was calculated. Abnormal values for both the rs-fMRI index and visuomotor reaction time were excluded if they fell outside the range of mean ± 2SD. Following this principle, we excluded the data of two athletes. This showed that the dALFF value in the rMCC brain region of the athletes was significantly positively correlated with the average visuomotor reaction time (*r* = 0.6403, *p* < 0.01), however, correlation was not present in the control group (Figure 5B). No other correlations were detected in the other brain regions.

## 4. Discussion

Herein, a joint static and dynamic analysis of ALFF and FC was performed in table tennis players, the results of which might lead to a more comprehensive understanding of intrinsic brain activity. Our results supported the proposed hypothesis that joint static and dynamic analysis is an appropriate approach to study the functional activity of table tennis players in the resting state. The two methods provided complementary and overlapping information. Notably, we found dynamic indicators is sensitive for the table tennis players in our study. Furthermore, we found that table tennis players exhibited alterations in intrinsic brain activity in visual, sensorimotor, and executive function-related brain regions. The visual and sensorimotor brain areas exhibited reduced local spontaneous brain activity and sFC, which were related to the high neural efficiency of visuomotor conversion due to long-term training and partly explains the faster visuomotor reaction time of the athletes. However, increased dFC was shown between the visual and executive function brain areas in the left hemisphere, which may be related to better executive abilities in athletes.

### 4.1. Complementary and overlapping results of static and dynamic analyses

Herein, the results of static and dynamic analyses were complementary and overlapping. Multiband sALFF and dALFF analyses revealed compared with the controls, the athletes exhibited a lower sALFF in the lITG and rMCC, whereas they had a higher dALFF in the rPoCG. We found that the dALFF of rMCC could predict the visuomotor reaction time of table tennis players. Regarding sFC and dFC analyses, differences were present between athletes and controls only when the lITG was used as the seed region. Significant differences were present in two brain regions in the sFC analysis and four brain regions in the dFC analysis. Both sFC and dFC analysis revealed changes in the FC between lITG and lIPG. In particular, the sFC values of the brain regions with group differences were smaller in athletes than in controls, whereas the dFC values in athletes were larger than those in controls (Gao et al., 2021). These findings suggest that the correlation between lITG and lIPG was weaker in athletes compared with that in controls at baseline, and that the fluctuations in their correlation coefficients over time were greater in athletes than in controls (Vahdat et al., 2011; Huang et al., 2018; Gao et al., 2021; Iraji et al., 2021; Zheng et al., 2021). Therefore, we speculate that the joint static and dynamic analysis can obtain complementary and overlapping results, enabling a more comprehensive exploration of brain activity than analysis by a single method. Notably, there are more dynamic results than static ones. We speculate two reasons for why dALFF and dFC outperformed sALFF and sFC in this study: first, it may be because we used the PerAF index when calculating the dynamic index, and the introduction of the percentage increases the sensitivity of the dynamic index (Chen et al., 2022; Gao et al., 2022). Second, dFC markers encode more behavioral information compared with static FC markers, such as processing speed and executive function (e.g., working memory and attention; Liégeois et al., 2019). Our dFC analysis detected changes in the FC of vision and frontal cortex brain regions, which are also key brain regions for executive function (Knöllner et al., 2022). Furthermore, the experimental data was obtained using 7T ultra-high field magnetic resonance, which has a higher temporal signal-to-noise ratio, and therefore 7T ultra-high field magnetic resonance may be suitable for the dynamic analysis of rs-fMRI (Hale et al., 2010). The above explanations remain speculation, and further experimentation of the same type is required to support this.

### 4.2. Relationship between the higher neural efficiency of the visual areas and faster visuomotor reaction time

Static and dynamic joint analysis revealed that table tennis players exhibited alterations in intrinsic brain activity in visual and motor function-related brain regions, with decreased cortical activity being the mainstay. Our behavioral results revealed that table tennis players exhibited a faster visuomotor reaction time than non-athletes. Previous studies reported that superior athletic performance in elite athletes may be associated with lower activity in some brain areas compared with non-athletes, consistent with the neural efficiency hypothesis (Del Percio et al., 2009; Neubauer and Fink, 2009; Guo et al., 2017; Danti et al., 2018; Li and Smith, 2021; Gao et al., 2023). This is consistent with our results. lITG is a high-level brain area of the ventral visual pathway, which is related to the position, color, posture, and facial recognition of objects. lITG is a brain region that is arguably central for visual object recognition in both humans and nonhuman primates (Li and DiCarlo, 2008; Jia et al., 2021). In a fMRI study of table tennis players on a visuospatial task, Guo et al. found that athletes exhibited lower brain activation in the regions of the left MTG, bilateral lingual gyrus, and lITG than non-athletes (Guo et al., 2017). Table tennis requires extensive visual participation. Players need to quickly receive and process various visual information, such as tracking the trajectory of the ball and observing the opponent’s hand and racket posture (Rodrigues et al., 2002; Hülsdünker et al., 2019; Piras et al., 2019; Shinkai et al., 2022). After long-term training, these athletes have formed a unique visual strategy, with the lITG function probably becoming more specialized, which is conducive to rapid response with higher neural efficiency (Yin et al., 2021).

dALFF analysis revealed that table tennis players also exhibited significantly reduced activity of rMCC. In a broad sense, the cingulate cortex is also a part of the SMN, which includes the midcingulate cortex (MCC; Mitchell et al., 2013; Herz et al., 2016; Vogt, 2016). The motor function of the MCC is critical and is mainly concentrated in the cingulate premotor area at the tail of the posterior MCC, which is involved in visual and spatial location and multisensory orientation of the head and body in space (Braak, 1976; Matelli et al., 1991). The neuron responses of the MCC were tuned for the force and direction of movement (Braak, 1976; Matelli et al., 1991). Table tennis is a fast racket sport. In addition to players requiring rapid visual processing, they need to accurately perceive the direction and make rapid direction adjustments to the body to hit the ball quickly and accurately (Rodrigues et al., 2002; Kaabi et al., 2022; Takami et al., 2022). Existing evidence shows that MCC receives information projections from the parietal lobe, especially the posterior parietal cortex (PPC) to regulate body orientation, reflect movement, and make rapid motor responses, through coordination with the supplemental motor area and connections to the spinal cord (Vogt, 2016). We found that the dALFF value of rMCC in table tennis players was significantly positively correlated with the visuomotor reaction time. A lower value means a shorter reaction time, which also proves that rMCC can quickly regulate visual movement with high neural efficiency, enabling athletes to have better performance.

sALFF and dALFF analysis provided the functional separation results of the brain regions. To study the functional integration of brain regions, we calculated voxel-wised sFC and dFC and found that the athlete group exhibited significantly lower sFC in lIPG and rMTG. lIPG is a part of the PPC that lies between the sensory and motor areas and is a multisensory cortical area that receives input from multiple sensory systems (including somatosensory, auditory, visual, motor, proprioceptive and vestibular signals; Grefkes et al., 2004; Whitlock, 2017). The PPC is a key brain region for sensorimotor translation and is involved in motor planning (Li et al., 2022). Table tennis players must frequently initiate targeted motor responses without extensive cognitive processing, and visual information must be quickly translated into motor commands. Information flows from the visual area to the PPC and then to the premotor and supplementary motor areas to complete the visuomotor transformation (Hülsdünker et al., 2018). We found that sFC decreased between lITG and rMTG. Tankus et al. demonstrated that the temporal lobe plays a role in transforming visual information into hand movements, and this connection may be linked to the dorsal pathway (Tankus and Fried, 2012; Breault et al., 2019). lITG is a high-level brain region of the ventral visual pathway, whereas MTG is involved in the perception of tools, body and hands, movement observation, and visuomotor (Lesourd et al., 2021; Amaral et al., 2022). The MTG helps predict self-generated movements by optimizing them based on the physics of the end effector, such as the hand or tool being used (Pazen et al., 2020). Table tennis places great emphasis on observing and predicting hand and racket posture. Through extensive training, players develop an efficient process for transforming visual input into rapid visual motor responses, allowing the execution of precise hand movements with ease. Thus, we found that table tennis players exhibited reduced cortical activity during several key processes of visuomotor conversion, which was consistent with the neural efficiency hypothesis. After long-term professional learning and training in table tennis, athletes exhibited high levels of automaticity in visual motor responses. Bassett et al. found that decreased integration between motor and visual modules was observed during motor skill-learning (Bassett et al., 2015). Decreased ALFF or FC in visual and sensorimotor brain regions reduces energy consumption to ensure that athletes can perform with high neural efficiency (Bertollo et al., 2020; Filho et al., 2021).

### 4.3. Changes of static and dynamic brain activity in brain areas related to executive function

Unlike the reduction in brain activity described above, table tennis players exhibited increased dFC when lITG was used as the seed region compared with controls. The observed functional changes in intrinsic brain activity in athletes *via* dFC analysis were primarily between the lITG and frontal cortex (lMFG, lSFGmedia, and lSFG). Traditionally, the frontal lobe is associated with executive function (Miyake et al., 2000; Cristofori et al., 2019). The middle frontal gyrus and superior frontal gyrus are two high-level cognitive brain areas located in the prefrontal cortex and involve executive functions, such as attention and working memory (du Boisgueheneuc et al., 2006; Li et al., 2013; Briggs et al., 2021). The lMFG is also considered a part of the ventrolateral prefrontal cortex (Kumar et al., 2022), which in primates receives visual information from the lITG (Romanski, 2007). The superior frontal gyrus includes SFGmedia and SFG, which are related to the cognitive control and default mode networks, and is strongly correlated with controlling and processing emotional/cognitive behavior (Li et al., 2013; Xu et al., 2022). In addition, the SFG plays an important role in the control and coordination of complex movements (Martino et al., 2011). In other studies involving athletes, changes in intrinsic brain activity were also found in the MFG, SFGmedia, and SFG. Elite snowboarding halfpipe athletes exhibited stronger activations in the MFG and SFG in response to incongruity when observing maneuvers; 8 weeks of Tai Chi Chuan exercise enhanced the functional connection between lMFG and the left superior parietal lobule; and soccer athletes exhibited increased brain activity in the bilateral MFG during motor control (Cui et al., 2019; Shi et al., 2020; Zhao et al., 2021). Visual information is critical to executive function. The lITG is a higher brain region of the ventral visual pathway (Knöllner et al., 2022). Increases in dFC from lITG to these three frontal brain regions (lMFG, lSFGmedia and lSFG) indicated dynamic instability and flexibility of the visual and executive areas in table tennis players. Therefore, the significant increase in dFC between lITG and the prefrontal cortex may be related to greater executive abilities, such as visuospatial working memory and visual selective attention in athletes (Huijgen et al., 2015; Wang et al., 2016; Elferink-Gemser et al., 2018).

Interestingly, regions with different cognitive and processing demands represent different levels of dFC. The brain networks/areas known to be involved in higher cognitive processing show a higher level of dynamism measured *via* dFC than that of networks/areas engaged in primary processing (Iraji et al., 2021). This may be a reason for the enhanced dFC between the lITG and prefrontal cortex.

## 5. Limitation of study

Initially, we did not categorize the table tennis players based on their current competition points or experience level. Previous studies reported that the changes of brain activity in athletes does not follow a linear pattern in relation to their training experience and skill level; however, we did not consider this during our experiments (Chang et al., 2018; Gao et al., 2021, 2023). We found changes of brain activity in regions related to executive function in athletes but did not administer an executive ability test before the study. This is an area that we can explore in future research. Notably, the time series in this study was relatively short, comprising only 160 volumes because of the limitations of fMRI acquisition (Tian et al., 2022). In future studies, it may be beneficial to increase this duration to more accurately evaluate the dynamic changes of the participants.

## 6. Conclusion

Herein, we found that joint static and dynamic analysis produced complementary and overlapping results in table tennis players. Therefore, we recommend this as an appropriate research method. When at the resting state, table tennis players exhibited a generalized reduction in ALFF and sFC in the visual and sensorimotor areas compared with those of the control group. In addition, we discovered a significant positive correlation between the athletes’ visuomotor reaction time and their dALFF in the rMCC. We speculate that table tennis players develop static and temporal dynamic changes in their brain function from long-term skill learning and training. These changes in brain activity enable them to achieve faster visuomotor responses, which aligns with the neural efficiency hypothesis. Our results further indicate that dFC from the lITG to prefrontal cortex regions, including lMFG, lSFGmedia, and lSFG, were enhanced in table tennis players. This finding suggests a potential correlation between improved executive abilities and the observed increase in connectivity.

## Data availability statement

The raw data supporting the conclusions of this article will be made available by the authors, without undue reservation.

## Ethics statement

The studies involving human participants were reviewed and approved by the ethics committee of Hangzhou Wuyunshan Hospital. The patients/participants provided their written informed consent to participate in this study.

## Author contributions

All authors listed have made a substantial, direct, and intellectual contribution to the work, and approved it for publication.

## Funding

This work was supported by grants from STI 2030 – Major Projects (2021ZD0201705), the Fundamental Research Funds for the Central Universities, and the Interdisciplinary Preresearch Project of Zhejiang University.

## Conflict of interest

The authors declare that the research was conducted in the absence of any commercial or financial relationships that could be construed as a potential conflict of interest.

## Publisher’s note

All claims expressed in this article are solely those of the authors and do not necessarily represent those of their affiliated organizations, or those of the publisher, the editors and the reviewers. Any product that may be evaluated in this article, or claim that may be made by its manufacturer, is not guaranteed or endorsed by the publisher.

## Footnotes

^1^http://restfmri.net/forum/restplus
