# Supplementary material for: Static and dynamic resting-state brain activity patterns of table tennis players in 7-Tesla MRI
Source: Front Neurosci. 2023 Jul 14;17:1202932. doi: 10.3389/fnins.2023.1202932 (PMC10375049; doi:10.3389/fnins.2023.1202932)
Supplement: Supplementary file 1 [file Presentation_1.pdf]

Supplementary Material

Static and dynamic resting-state brain activity patterns of table tennis players in 7-Tesla MRI

Yuyang Li[1 †], Mengqi Zhao[2,3 †], Yuting Cao[1,4], Yanyan Gao[2,3], Yadan Wang[5], Bing Yun[6], Le Luo[7], Wenming Liu[8*], Chanying Zheng[1,4*]

*** Correspondence:**
Chanying Zheng, zhengchanying@zju.edu.cn

Wenming Liu, liuwenming@zju.edu.cn

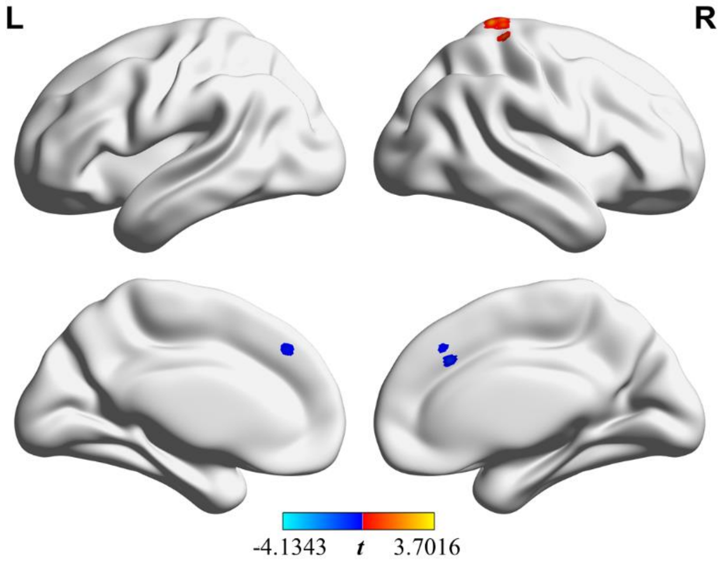

Supplementary Figure 1. dALFF differences between athletes and controls on the basis of coefficient of variation (CV) metric. Two-sample t-test, voxel-level p < 0.01, cluster-level p < 0.05, GRF correction; Blue area: Athletes < Controls. Red area: Athletes > Controls.

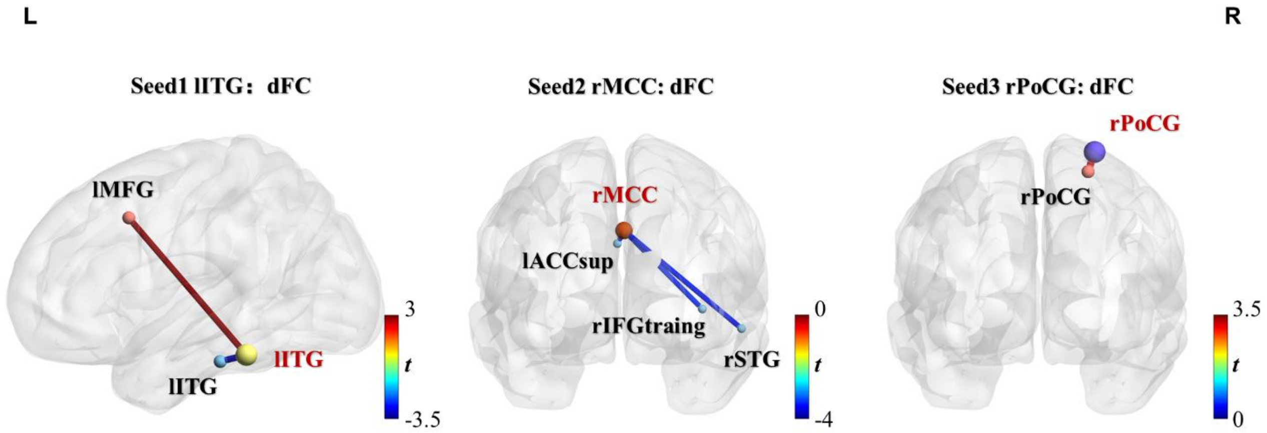

Supplementary Figure 2. dFC differences between athletes and controls on the basis of coefficient of variation (CV) metric. The significantly increased dFC in athlete group was marked in warm colors, while the significantly decreased dFC was marked in cold colors. The colors of the lines represent t-values. Two-sample *t*-test, voxel-level $p < 0.01$, cluster-level $p < 0.05$, GRF correction. dFC, dynamic functional connectivity; lITG, left inferior temporal gyrus; lMFG, left middle frontal gyrus; rMCC, right middle cingulate & paracingulate gyri; rSTG, right superior temporal gyrus; rIFGtraing, right inferior frontal gyrus-triangular part; lACCsup, left anterior cingulate cortex-supracallosal; rPoCG, right postcentral gyrus.

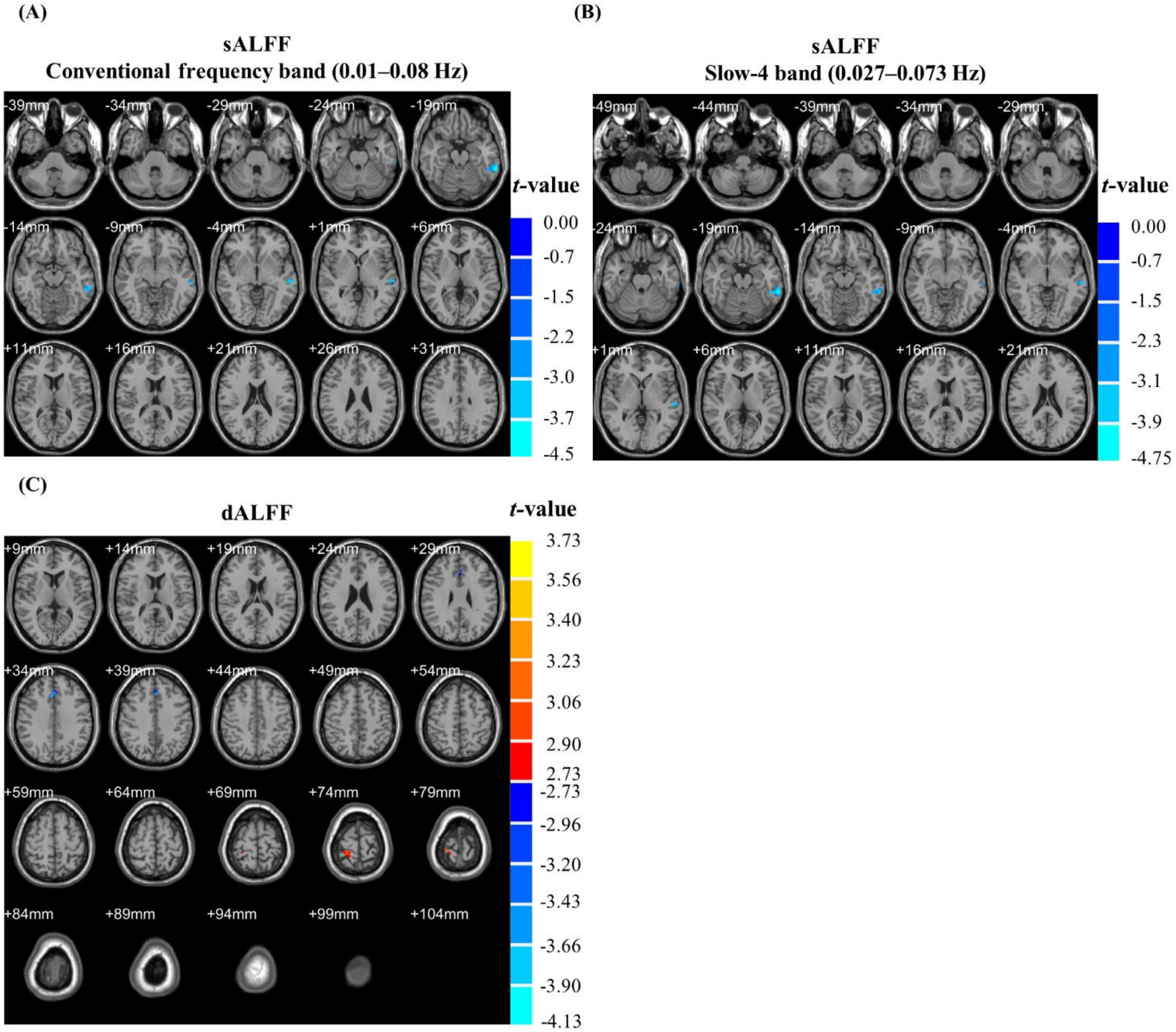

Supplementary Figure 3. Axis map of the sALFF (in two frequency bands) and the dALFF (defined by PerAF) differences between athletes and controls. (A) The sALFF differences in the conventional frequency band (0.01–0.08 Hz) between athletes and controls; (B) The sALFF differences in the slow-4 band (0.027–0.073 Hz) between athletes and controls; (C) The dALFF differences between athletes and controls. Two-sample *t*-test, voxel-level $p < 0.01$, cluster-level $p < 0.05$, GRF correction; Blue area: Athletes < Controls. Red area: Athletes > Controls.

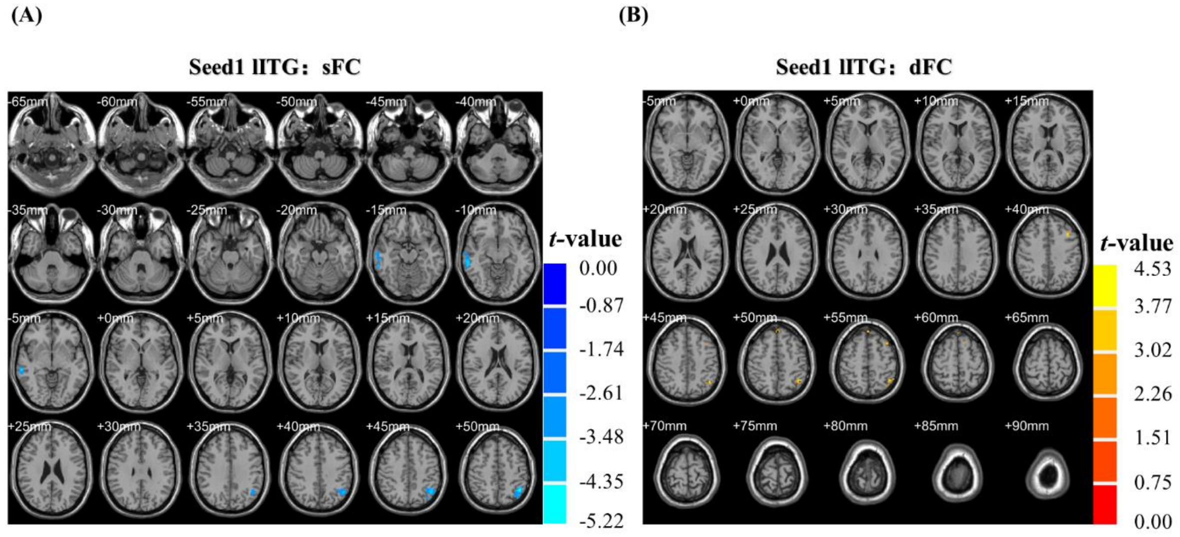

Supplementary Figure 4. Axis map of the lITG seed-based static and dynamic FC differences between athletes and controls. Significantly increased sFC or dFC in the athlete group is shown in warm colors, whereas significantly decreased sFC or dFC is indicated in cold colors. The colors of the lines represent t-values. Two-sample *t*-test, voxel-level $p < 0.01$, cluster-level $p < 0.05$, GRF correction. sFC, static functional connectivity; dFC, dynamic functional connectivity; lITG: left inferior temporal gyru; Blue area: Athletes < Controls. Red area: Athletes > Controls.
